# Anti-stigma narratives and emotional comfort against health crisis: a context analysis of UGC short videos from patients with COVID-19 infections

**DOI:** 10.1038/s41598-023-41184-4

**Published:** 2023-09-07

**Authors:** Lin Tan, Qing-yi Wang, Qiu-ju Zhang

**Affiliations:** 1 School of Marxism, Xi’an Jiaotong University, Xi’an, 710049 China; 2 College of Basic Medical, Fourth Military Medical University, Xi’an, 710032 China

**Keywords:** Health policy, Health services, Public health

## Abstract

Patients narratives are being recorded increasingly frequently and spontaneously in short user produced content (UGC) films, which may have an impact on the vlogger’s health as well as the public’s comprehension of the relevant health concerns. This paper addressed three research questions regarding the population characteristics of UGC video publishers, the narrative theme of the videos, and the emotional orientation of the commenters. This study aimed to deepen our understanding of COVID-19 patients’ narrative intentions and emotional needs through the theoretical frameworks of theory of planned behavior (TPB) and negative dominance theory (NDT). We collected 335 videos from 28 COVID-19 patients and 572,052 comments as samples on Douyin platform, the largest short-video website in China. Using Latent Semantic Analysis, we analyzed the descriptive information of the video blogs, the narrative textual information of the videos, and the emotional orientation of the comments. Our findings revealled seven categories of narrative themes, with 52.1% of video comments exhibiting a positive emotional orientation. Within a framework integrating TPB and NDT theories, we analyzed the behavioral intentions of vloggers and viewers during COVID-19 epidemic, and interpreted the persistent posting of videos and the active posting of comments as positive actions that counteracted the multiple effects of negative messages. This study contributes to the understanding of individual narratives in macro-risk communication, both theoretically and empirically, and offers policy recommendations in relevant fields.

## Introduction

The 2019 coronavirus disease (COVID-19) has emerged as a significant public health threat. After spreading extensively in numerous countries subsequently, the World Health Organization (WHO) declared the COVID-19 an international health emergency raising to a global pandemic in March 2020^1^. The pandemic was considered as the most crucial global health calamity of the century^2^. Until July 26, 2023, WHO had reported a total of 768.5 million confirmed cases and over 6.8 million deaths due to COVID-19 worldwide^3^.

The COVID-19 epidemic had a multifaceted impact on people’s lives, particularly in relation to media communication. COVID-19 increased people’s daily exposure to emerging media, as they tend to share information and exchange feelings rather than have face-to-face interactions. Furthermore, spatial limitations, by increasing loneliness and anxiety, might change communication behaviors and habits, and affect social relationships, which are the key elements to human well-being. Within these contexts, the use of digital technologies had been recommended to get epidemic prevention knowledge as well as relieve pressure and loneliness^4^. The same was true in a survey of over 6000 adults in United States, social media had been widely used for health-related purposes, such as learning about COVID-19^5^. With greater trust in data from health professionals, academic institutions, and government agencies, people may also share COVID-19 videos with families and friends to cope with anxiety, anger, and fear^6^.Multiple cross-sectional surveys had shown that social media was increasingly used as a powerful communication tool^7,8^, and media played a key role in influencing social anxiety during the COVID-19 epidemic^9,10^, but few studies viewed social media as an information hub for building social connections center^11^ and explored its role in public health crises by analyzing media narrative texts.

In modern society, the media plays a critical role in perpetuating stigma, whether by creating it^12^, addressing it^13^, or measuring its impact^14^. Excessive COVID-19 information in the social media might exacerbate the stigmatization of infected individuals, e.g. cyberbullying, stigmatization, polarization of public opinion, and other forms of crime^15^. By analyzing the COVID-19-related tweets, it was very obvious that media have been suffused with news, messages, videos, hashtags and meme of the COVID-19 pandemic, which seem like the awareness about COVID-19 spread like the pandemic itself^16^. According to Erving Goffman’s(1963) classification of stigma, COVID-19 falls into the group based on physical deformity. In the complex social discourse of modernity, the metaphor of illness (e.g. COVID-19) revealed a punitive notion in the complex social discourse of modernity, where illness was used as a sign of evil, as a sort of symbol to identify the punished person or group^17^. The portrayal of diseases in the media, such as AIDS and mental illness^18^, exemplified the stigmatization targeted at specific groups^19^. Previous studies had investigated the stigma resistance of diseases using group or semi-structured interviews^20,21^, which were more easily influenced by the interview environment and the personality of the interviewee. While some studies have analyzed text messages from health forums to examine stigmatization of obese patients^22^, the intensity, duration, and population involved differ significantly when compared to the stigma surrounding COVID-19.

Based on the above research background and literature review, we founded that research on illness stigma mostly uses questionnaire analysis to capture the macro situation, lacking attention to the active narrative of stigmatised individuals, as well as research on social dialogue between others and them. We therefore proposed the following research questions:

**RQ1**: What is the overall profile of vloggers who produced these COVID-19 videos, and what are the features of these vlogs?
**RQ2**: What are general landscape and focal topics of the relevant UGC videos ?
**RQ3**: How do viewers cognitively and affectively respond to these self-narrative videos?

## Methods

### Theory of planned behavior (TPB)

The TPB proposed by Ajzen^23^ is a highly influential theory in social psychology that explains individual behaviors. It assumed that people’s actual behaviors were directly influenced by behavioral intentions, which in turn, resulted from comprehensive evaluations of attitudes, subjective norms, and perceived behavioral control. The TPB predicted that the more favorably an individual evaluated a particular behavior, the more likely he or she would intend to perform that behavior^24^. Subjective norm reflected a person’s perception that the more an individual perceived that significant others think he or she should engage in the behavior, the greater an individual’s level of motivation to complied with those others^25^. Internal subjective norms refered to influences from friends, family, coworkers, etc., while external ones came from others, e.g. social networks^26^. Perceived behavioral control reflected perceptions of internal and external constraints on behaviors. The TPB had been widely proven to be an effective theoretical framework for understanding various health issues^27^. Specifically, it has been found to have better predictive effects on behaviors related to different COVID-19 issues^28^, e.g. telecommuting^29^, receiving vaccine^30^, paying for health live streaming^31^.

### Negative dominance theory (NDT)

The NDT can help explain attitudinal and perceived behavioral control in risk communication. It describes how individuals process negative and positive information, particularly in high-concern situations^32,33^. In general, negative information carried significantly more weight in terms of its impact on the people involved. They expressed emotions more intensely about losses than gains^32^. This theory could rationalize the high level of concern and sensitivity to relevant information during the quarantine period due to COVID-19 infection. A person at health risk was accustomed to prioritizing access to negative news, which required an abundance of positive news to counteract. With social media at their disposal to voice their opinions, the perceived behavioral control they had could motivate them to take action to mitigate real risks and psychological concerns they faced.

### LSA analysis

The context analysis is acknowledged as a tool for delving into the deeper meanings of words^34^. Latent Semantic Analysis (LSA), a commonly used natural language processing (NLP) tool, is a specific method for extracting meaning of words in the contextual-usage by the tools of computational science^35^. With advances in computer-assisted content analysis, it is more conducive for social scientists to explore the process and significance of important events from the vast amount of textual sources (e.g., archives, newspapers, videos), organized in complex data structures^36^. In this paper, the content analysis method was employed based on LSA.

### Data source

This study used the data of UGC videos from Douyin, the most extensive video sharing social network service in China, with up to 920 million active users. After manual searching of the platform and handworked merging of synonyms in a pre-pilot study, ten hashtags (e.g., # quarantine diary, #self-care, #COVID-19 records) compiled by the research team in the pilot study were used to identify appropriate videos. First, we randomly scraped over 1000 publicly available clips of 465 vloggers and classified them under the subjects of patients’ narrative, using an unofficial Application Program Interface tool. After the data collection, the initial types were created, and the entire video corpus were established according to the following rules: The selected samples should be (1) with clear voice and logical expression, (2) longer than 30 s and shorter than 10 min, (3) assembled in quarantine diary collection with more than 3 videos, (4) followed by more than 1000 viewers, (5) with clear location information. Finally, in the script theme analysis, 335 short videos and their metadata from 28 UGC vloggers were collected to create our research data set. The associated metadata contained ID, vlogger’s profiles, video texts, number of fans, number of likes. And in the emotional analysis of comments, 572,052 comments from the above video were used as analyzed data.

### Design

**Figure 1:**
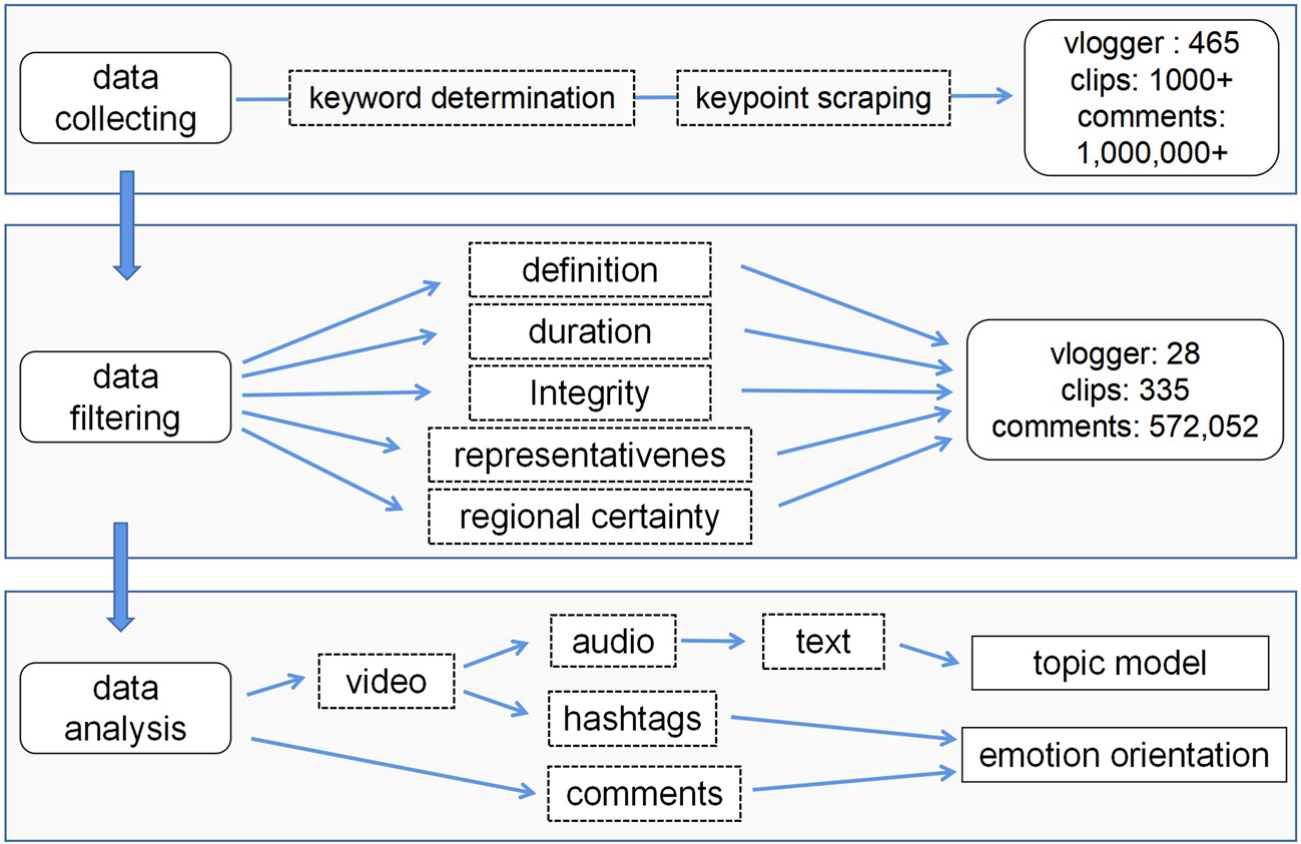
Experimental design and data processing procedures.

The experimental design and data processing is shown in Fig. 1. We used Python programming for data crawling and analysis, and the *pyppeteer* toolkit for comments grabbing. The video scripts of samples were extracted using speech recognition and manual correction. All scripts as well as comments were split using the *jieba* package, and a user-defined dictionary was used in the splitting, with special addition of common words related to the epidemic, etc. Then, we filtered the results based on the stop word table of Baidu, HIT and Sichuan University^37^, and supplemented stop words commonly found in Douyin.

To conduct thematic analysis of the scripts, we used the *sklearn* toolkit for TF-IDF algorithm calculation^38^, which extracted 1000 keywords and created a word frequency matrix. Within this matrix, we performed word frequency statistics and word cloud plotting for the top 100 keywords. The top 30 keywords were extracted and displayed using the *PyLDAvis* package, a web based interactive topic model visualization^39^.

The Affective Lexicon Ontology (ALO), an modified version of Ekman’s six-category emotion classification, was used to classify the comment contents into 7 sentiments^40^, we determined the sentiment color of each comment by matching the Chinese word segmentation results with the ALO library. Each word corresponds to a polarity under each category of emotion. Where 0 represented neutral, 1 represented positive and 2 represented negative.We statistically calculated the sentiment orientation of the entire comment based on the scores of the Chinese words in each comment and thus classified the whole comment text into positive, neutral, and negative categories.

## Results

### Descriptive statistics for vloggers, videos, and comments

The data of the 28 vloggers in the sample and their short videos were shown in Table 1. They were all native Chinese speakers. 17 vloggers were living in China. 75% had a young-looking appearance, and gender ratios were close to 1:1 (male=13, female=15). 78.6% vloggers introduced their professionals or social identities in videos, with top 3 occupations being undisclosed (n=6), the Internet celebrities (n=5), and students (n=3). They had 1,543,956 fans on average.

**Table 1:** Features of 28 vloggers and their 335 short videos.

	Living area	Occupation	Gender	Age category	Number of fans	Number of samples	Number of comments
#1	England	Student	F	Youth	460,945	11	25,408
#2	USA	Internet celebrity	F	Youth	640,865	5	14,087
#3	USA	Undisclosed	M	Middle-aged	135,339	13	6,888
#4	USA	Housewife	F	Middle-aged	160,321	16	24,626
#5	France	Undisclosed	F	Youth	60,840	5	1,964
#6	German	Teacher	M	Youth	157,758	11	5,809
#7	Australia	Nurse	M	Middle-aged	640,756	8	5,860
#8	Austria	Housewife	F	Middle-aged	1,129,890	16	6,960
#9	Japan	Undisclosed	M	Middle-aged	8,383,758	17	295,211
#10	Japan	Freelance	M	Youth	5,100,468	5	28,333
#11	Korea	Internet celebrity	F	Youth	4,198,240	4	12,685
#12	China	Actor	M	Middle-aged	3,065,098	5	17,760
#13	China	Office worker	F	Youth	1,439	13	648
#14	China	Undisclosed	M	Middle-aged	3172	15	705
#15	China	Ballerina	F	Youth	4,201	8	1,057
#16	China	Doctor	M	The old	420,123	9	2,660
#17	China	Internet celebrity	F	Youth	10,296,908	9	46,018
#18	China	Student	M	Youth	2,304,002	8	24,666
#19	China	Internet celebrity	F	Youth	2,907,143	25	8,891
#20	China	Director	M	Middle-aged	10,758	15	6,850
#21	China	Teachers	M	Youth	2,584,945	12	5,333
#22	China	Athletes	F	Youth	198,397	32	12,749
#23	China	Researcher	M	Middle-aged	300,767	18	9,792
#24	China	Undisclosed	F	Youth	19,395	23	1,096
#25	China	Media staff	F	Youth	26,758	7	2,859
#26	China	Student	F	Youth	1,038	8	174
#27	China	Internet celebrity	M	Youth	3,069	4	205
#28	China	Undisclosed	F	Youth	14,385	13	2,758
	Totally				43,230,778	335	572,052

The 335 videos had an average duration of 167 seconds, with a standard deviation of 170 s. The number of videos in the collection was 11.96 ± 6.77, and the average number of comments per vlogger was 20430.4 ± 54949.4. The results indicated that comments correlated significantly with fans (r = 0.632, *p*< 0.01). However, the correlation coefficient value between samples and fans was – 0.100, and the *p*-value was 0.614> 0.05, thus there was no correlation between samples and fans.

### Video topics and narrative intentions

**Figure 2:**
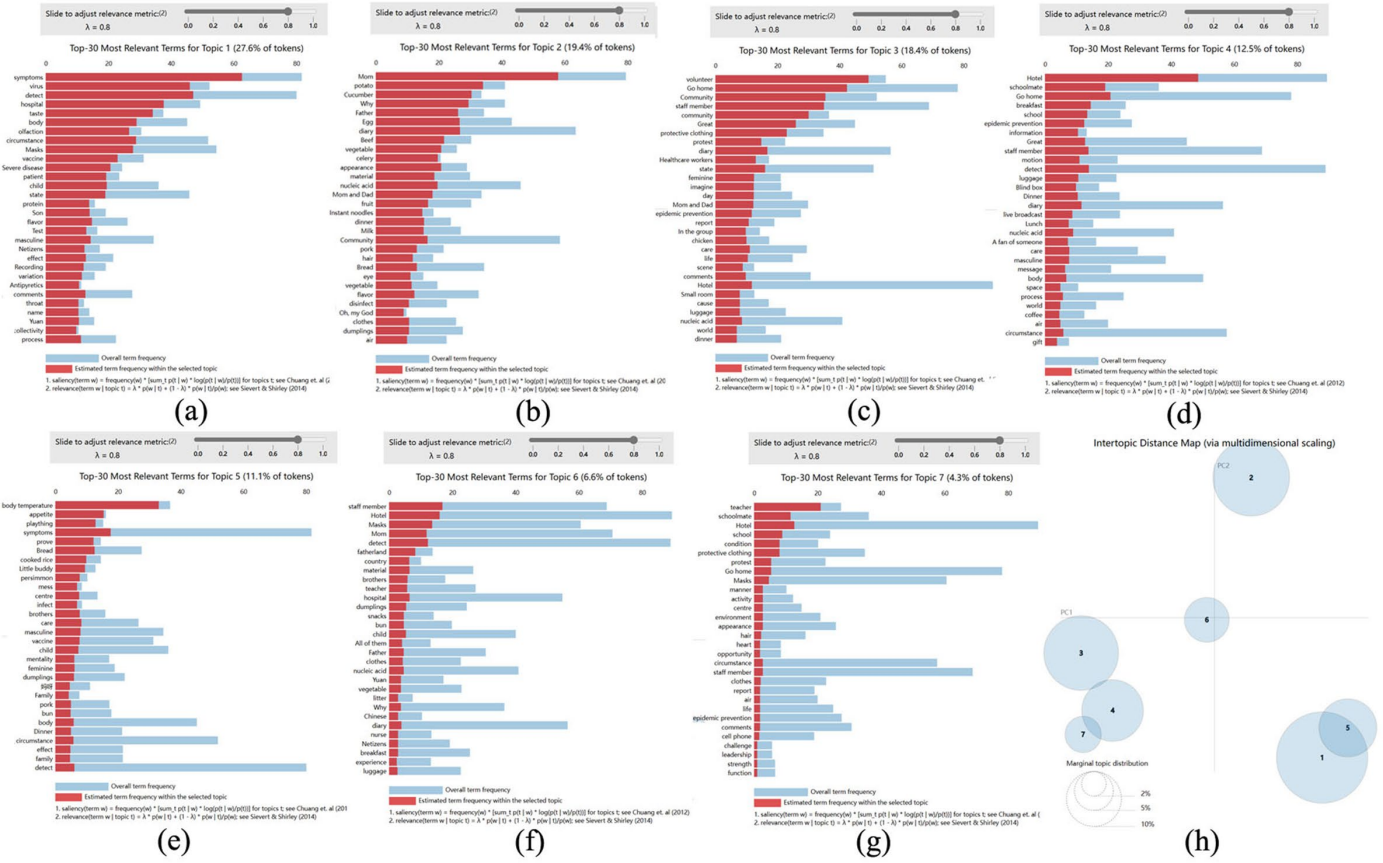
7 themes and keywords of the short videos.

The optimal number of topics was determined to be 7 using the perplexity index, as is shown in Fig. 2. The topics and their respective proportions were listed as below: symptoms and feelings ( 27.6% ), food and medical care (19.4%) , medical professionals and volunteers (18.4%), living atmosphere comparison (12.5%), family and friends (11.1%) ,country and race(6.7%) and campus life(4.3%).

Topic 1, shown in Fig. 2a, contained the following subject terms, symptoms, detect, virus, hospital, taste, body, patient, flavor, mess, etc. Almost all vloggers (n=27) described specific symptoms and emphasized personal feelings from first-person narrative. 67.9% vloggers, as epidemic risk-takers, cited or commented on information/predictions about the COVID-19 from traditional media and medical agencies.

Topic 2, shown in Fig. 2b, involved terms such as mom, diary, egg, father, vegetable, material, dinner, milk, instant noodles, bread, clothes, etc. Vloggers mainly expressed concern about the limited supplies in the quarantined state, or their joy at the abundance of supplies after shopping/distribution, probably because food reserves are associated with family warmth in traditional Chinese culture. 82.1% vloggers expressed directly that they missed their parents deeply while they were ill.

Topic 3, shown in Fig. 2c, had a coverage of volunteer, community, go home, staffs, protective clothing, healthcare workers, epidemic prevention, feminine, etc. The vloggers mainly introduced hard works of medical staff and volunteers, as well as rescuing and soothing, and even directly expressed sincere thanks and heartfelt praise to them.

Topic 4, shown in Fig. 2d, included hotel, schoolmate, go home, breakfast, information, epidemic prevention, motion, blind box, luggage, dinner, space, coffee, etc. By comparing some similar life moments, the vloggers described the difference in conditions and moods between the quarantined life and the previous life, expressing their dissatisfaction with the current situation and the desire to return to a normal life.

Topic 5, shown in Fig. 2e, covered the items of companionship, little buddy, concern, masculine, brother, meals, infection, mindset, dumpling, family, etc. The vloggers expressed concern and longing for their friends and family, and some of them referred to fans as family, encouraging them to get through the tough times.

Topic 6, shown in Fig. 2f, comprised items such as fatherland, country, hometown, brothers, milk tea, Chinese snacks, vaccine, dumplings, child, etc. Most of these vloggers praised the public health system for its accuracy and effectiveness in responding to the epidemic.

Topic 7, shown in Fig. 2g, included teacher, schoolmate, condition, protective clothing, activity center, environment, challenge, leadership, etc. Most of these were related to the campus life.

Figure 2h showed the intertopic distance map of the above 7 topics.

### Viewers’ emotional response and featured videos

**Figure 3:**
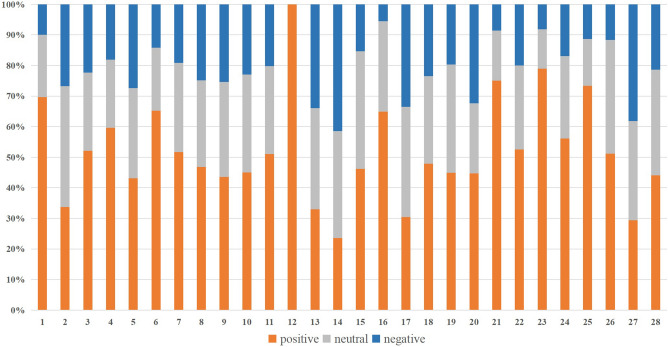
The emotional orientations of comments.

The emotional orientations of 572,052 comments were calculated by machine learning methods, and the results sorted by vloggers were shown in Fig. 3. The emotional orientations of all comments were counted, and the proportion of positive, neutral, and negative emotions were 52.1%, 27.5%, and 20.4%, respectively, proving that most people provided emotional social support to the patients. The comments were grouped according to 28 vloggers, and the emotional tendencies of the comments were counted according to positive, neutral, and negative as shown in Fig. 3. Among them, 24 vloggers had more positive than negative comment emotion. Three of the remaining four vloggers had a small number of followers, and their interactions with their fans were irregular, unstable, and detached, resulting in more neutral comments and fewer positive comments. Another vloggers had a more entertaining narrative style, which might have reduced fan empathy. The emotional score is 0.631±0.123, indicating a neutral to positive emotional tone. Chronologically, the change of netizens’ emotions towards different videos of the same vlogger was obscure. Comparing the emotions of comments on the first/last clip, it was noted that the enhanced attention and support burnout were not apparent.

According to ALO, the emotion classification and percentage of comments were as follows: nice (including respect, praise, believe, like, wish, and other subcategories) accounting for 56.74%, depressed (including sadness, disappointment, guilt, missing) accounting for 14.78% , happy (including joy, comfort) accounting for 12.92%, disgusted (including annoyed, derogatory, jealous, suspicious) accounting for 11.05%, fearful (including flustered, scared, ashamed) accounting for 3.70%, anger accounting for 0.43%, and surprised accounting for 0.38%.

## Discussion

Through the above research, we had summarized some research findings as follows:

To answer RQ1, we compared and analysed the personal information and types of videos published by the vloggers. First, we found that infected individuals labelled as morally deficient may be perceived as failing in their duty of self-management and thus posing a threat to public health. In a public health crisis, they all needed social support and there were no significant differences based on geography, class or gender. When in quarantine, social support from family and friends was limited, and they relied heavily on social media (e.g. short videos) to stay in touch with society, and posting short UGC videos was an active behaviour under conditions of their perceived behavioural control. Second, although they expressed concern about the unknown damage of the virus and dissatisfaction with the hardship of life caused by the epidemic, their proactive behaviour of posting videos indicated their autonomous will to obtain additional social support instead of enduring the disease alone. By posting short videos revealing their faces and scenes from their lives rather than descriptive text, they showed their sincerity and honesty in being willing to let others listen, and also indicated that they did not consider infection with COVID-19 a shame to be discussed in public cyberspace. Third, the increasingly convenient and efficient Internet services are changing the original status of medical personnel, family members and friends as the main sources of social support for medical affairs^41^. The sharing of certain medical conditions and the expression of opinions on health issues in public spaces is becoming more common, which effectively complements the authoritative information disseminated by official institutions^42^.

For RQ2, we performed textual analysis. The central themes of these narratives were still their personal lives under the influence of the epidemic,and the narrative perspectives,themes,and approaches were clearly personalised. Even many vlogs continued the original content type and production style. The vloggers successfully played a social role in rebuilding a sense of dignity, belonging, and value, and simultaneously they demonstrated their personal values in promoting mutual aid among patients. For example,a doctor directly explained medical knowledge and offered free consultations on social media.This is a further indication of the narrative value that distinguishes UGC from occupationally-generated content (OGC). We also found that the narrative style of UGC videos differed significantly from government health policy promotion^43^. However, many vloggers used official information and opinions as material or evidence in their videos, e.g. reminding netizens to focus on epidemic prevention. A minority of vloggers (21.4%) re-disseminated expert reports in their videos, while most (85.7%) preferred to describe personal experiences and feelings. In other words, the risk was more about “what it was portrayed as” than what it literally was. In this sense, this study confirmed the “constructivism of risk” in the health domain^44^. After the vloggers recovered, they rarely posted videos about their experience of quarantine. We also confirmed that the TPB could explain individual information dissemination behaviour in public health crises.

Regarding RQ3, we further investigated the role and effect of emotions in social risk communication by analysing the comments. First, the videos of COVID-19 inspired a large number of comments, which may be explained by the strong correlation between images and emotional stimuli^45^, in addition to being consistent with the NDT. Videos, due to their visual nature, were more closely associated with emotions than texts^46^, and could have the potential to play a positive role. Second, emotions not only have a communicative and encouraging effect, but can also have other complex effects. Emotional resonance, which can easily arouse public concern, could appeal to individuals to make concerted efforts, thus stimulating social mobilisation^47^. On the other hand, it should be noted that in the process of generation, interaction, aggregation, alienation and decay of emotions, some small amounts of negative emotions tend to decay, expand and polarise^48^. Third, emotional support is a manifestation of understanding the other person’s experience, usually presented in the form of comfort and encouragement, which has a very prominent effect in promoting the establishment of people’s self-esteem^49^. This recognition of others’ narratives could have a positive impact on their health^50^: recognizing the validity of emotions is an important practical requirement in public health risk communication^51^.

## Conclusions

In this paper, we analysed the audience characteristics and narrative theme of UGC short videos, and the emotional orientations of relevant comments during the COVID-19, in order to explore in more detail individuals’ attitudes towards stigma, stigma resistance behaviour and social support in the health crisis. We found that they were willing and proactive in posting relevant narratives on social platforms in order to resist stigmatisation of their group by others, as well as to receive informational and emotional support. This study presented a structured analysis and visualised the UCG content using data mining technology. It improved the macro-level research methods by using textual analysis of personal narratives in addition to statistical analysis. Furthermore, it supplied guidance for the institutions in developing health communication strategies. More emphasis, for example, should be placed on the role of scientists, media professionals, businesses, and ordinary citizens in risk dialogue, which helps to strengthen institutions’ attention to empowering the media on public health issues^52^ and incorporating media factors into the formulation of health promotion policies. Meanwhile, the media should pay attention to the changes in the distribution of “gatekeepers” role^53^ ,and jointly work with health authorities to restrict information fabrication for “eye-catching” purposes^54^ by means of algorithm design, content validation and user ratings^55^, thus preventing public health crises from getting entertained.

## Limitations and future directions

This study has several limitations that could be continuously improved in subsequent studies. Firstly, this paper focuses only on a single platform, so the narratives patterns can be limited. Secondly, the videos and data in this paper is analyzed by context analysis method, without accounts from vloggers about their motivation, therefore questionnaires and semi-structured surveys could be supplemented in future studies. Thirdly, due to the limitation of the software package, it is not possible to analyze comments and re-comments separately, and their hierarchical analysis by using social network analysis can be used as a follow-up study for this paper.

## Additional information

Data are obtained from Douyin website https://www.douyin.com/ (accessed on 9 November 2022). From an ethical perspective, the anonymity of the vloggers and commenters has been preserved.

## Data Availability

The datasets generated during and/or analysed during the current study are available in the OSF repository, (https://osf.io/download/6402328c40cecd0c5f76e194/).
